# Supplementary material for: Resistance and heteroresistance as a consequence of colistin therapy during Acinetobacter baumannii murine pneumonia
Source: bioRxiv. 2025 Jul 24:2025.07.24.666669. Preprint. [Version 1] doi: 10.1101/2025.07.24.666669 (PMC12330716; doi:10.1101/2025.07.24.666669)
Supplement: Supplementary file 1 [file media-1.pdf]

SI Figures and Tables

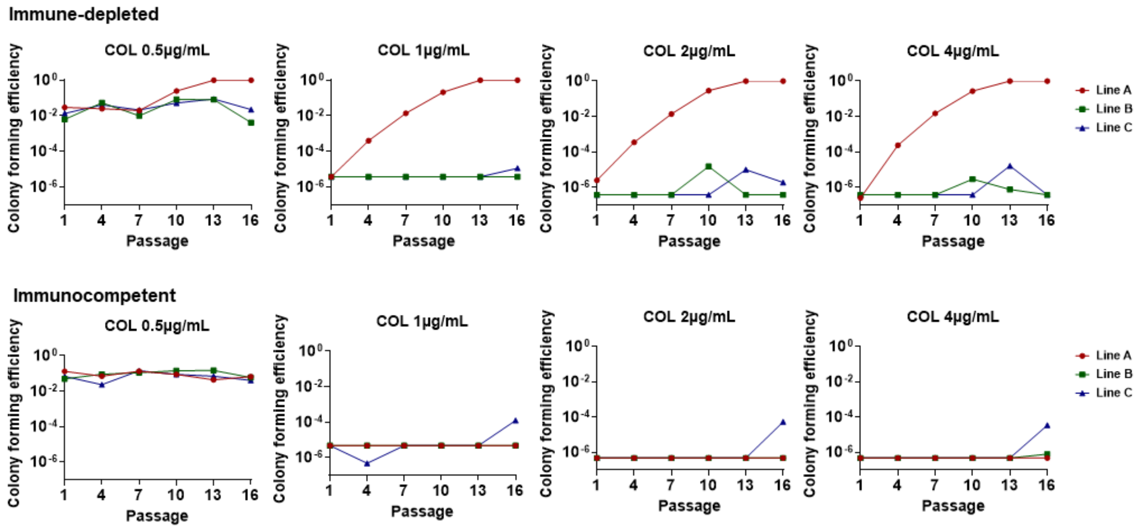

Fig. S1. Quantification of mutants with altered colistin susceptibility in passaged pools.

Frozen pools from each passage were incubated on LB agar containing 0 or 4 μg/mL colistin. CFE was calculated as described (Materials and Methods). Colonies appearing above baseline CFE were re-streaked on identical colistin-containing plates and saved for analysis (Dataset S1). The COL 4μg/mL results are identical to the data shown in Fig.1D-E and redisplayed here.

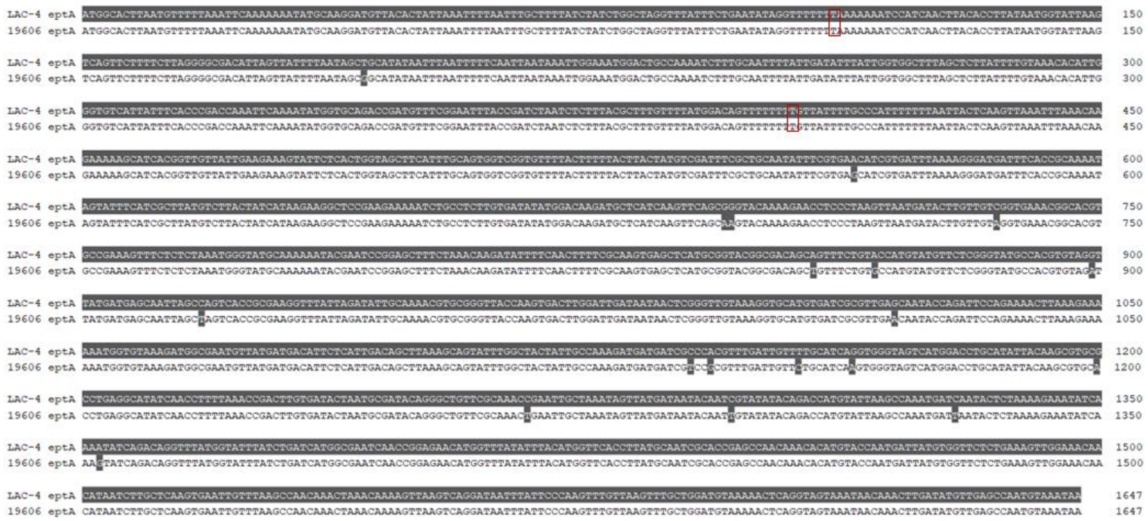

Fig. S2. Alignment of LAC-4 and ATCC19606 *eptA*.

The ATCC19606 *eptA* gene was mapped to the LAC-4 chromosome using Geneious Prime which shows 98.846% identity to this region. The LAC-4 reference genome had two missing T bases (red squares) at position 111 and 405/1647 causing this region to be annotated as two overlapping ORF's (ABLAC_18270-18280). WGS confirmed the presence of the T bases in the strains used in this study.

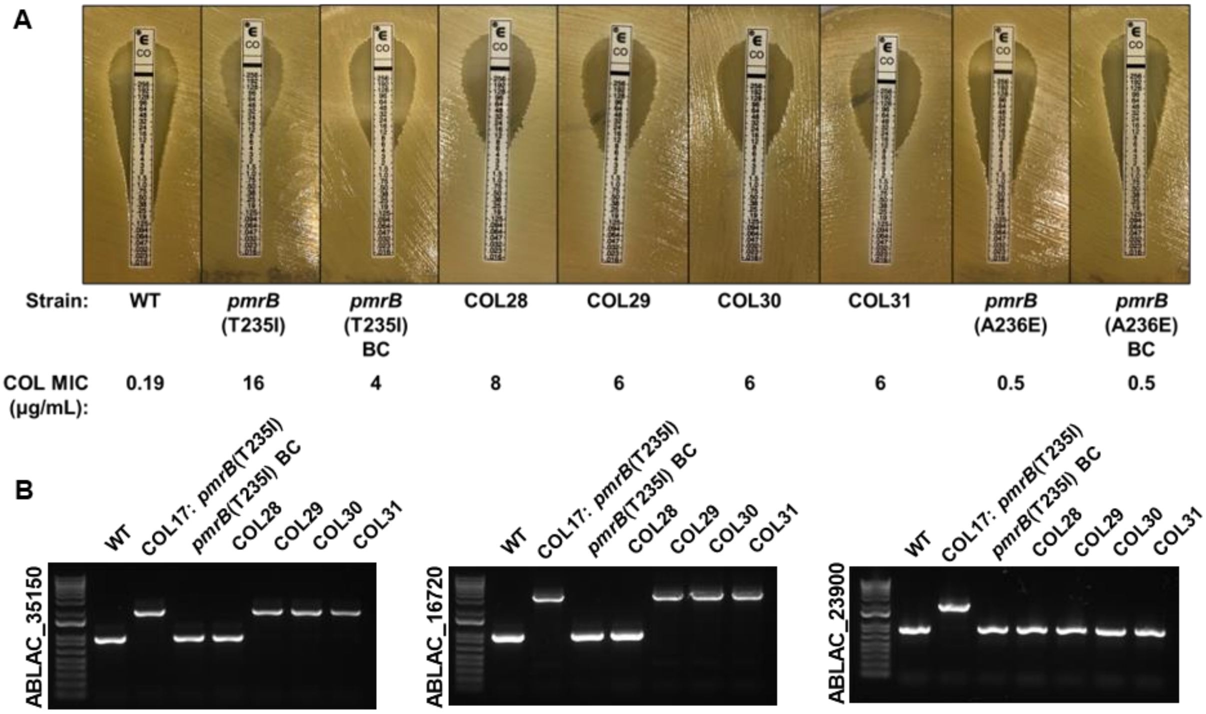

Fig. S3. The *pmrB*(T235I) and IS*Aba1*::ABLAC_23900 mutations are associated with high-level colistin resistance.

A. Susceptibility of isolates with the *pmrB*(T235I) mutation to colistin. The backcrossed (BC) strains were generated via homologous recombination (Materials and Methods) and the COL28-31 isolates emerged during the lung passages (Dataset S1). Colistin MIC determination via E-test strip. MIC determined as described (Materials and Methods). **B.** PCR amplification of the denoted genes in isolates with the *pmrB*(T235I) mutation. ABLAC_35150 encodes a putative thioesterase, ABLAC_16720 encodes an asparagine synthase and ABLAC_23900 encodes a putative TetR family transcriptional regulator. *Acinetobacter baumannii*

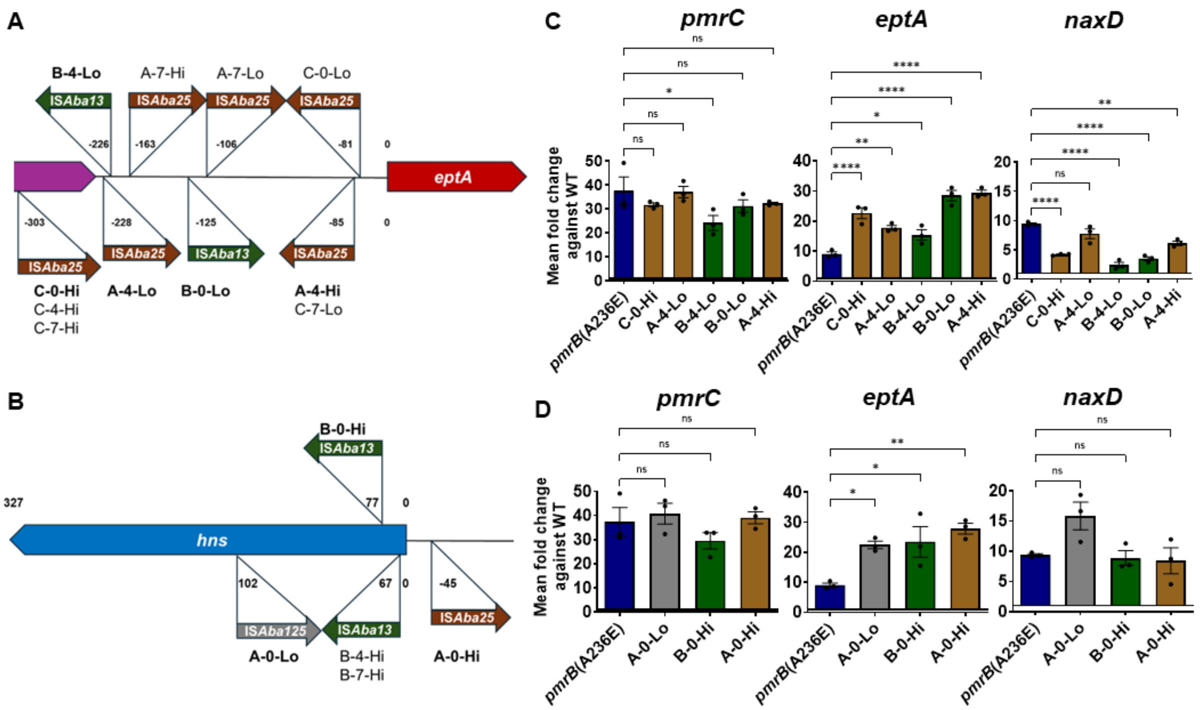

Fig. S4. IS*Aba* insertions in the vicinity of *eptA* or *hns* do not increase *pmrC* or *naxD* expression.

A-B. Location and orientation of IS*Aba* insertions in colistin-enriched *pmrB*(A236E) isolates. Figures not drawn to scale. **C-D.** Expression of genes that modify lipid A in the *pmrB*(A236E) isolates. Representative isolates (in bold) were selected for further analysis. Transcript levels of *pmrC, eptA and naxD* in mutants relative to WT was quantified via RT-qPCR. Displayed are mean ± SEM from three biological replicates shown. **A-D.** Isolate nomenclature refers to “Line – Day collected – Level of colistin exposure at day 0”. **A-D.** The schematics and *eptA* data are the same shown in Figure 5. **C-D.** Statistical analysis of gene expression changes were performed using one-way Anova followed by Dunnett's multiple comparison. *$P < 0.05$, **$P < 0.01$, ****$P < 0.0001$.

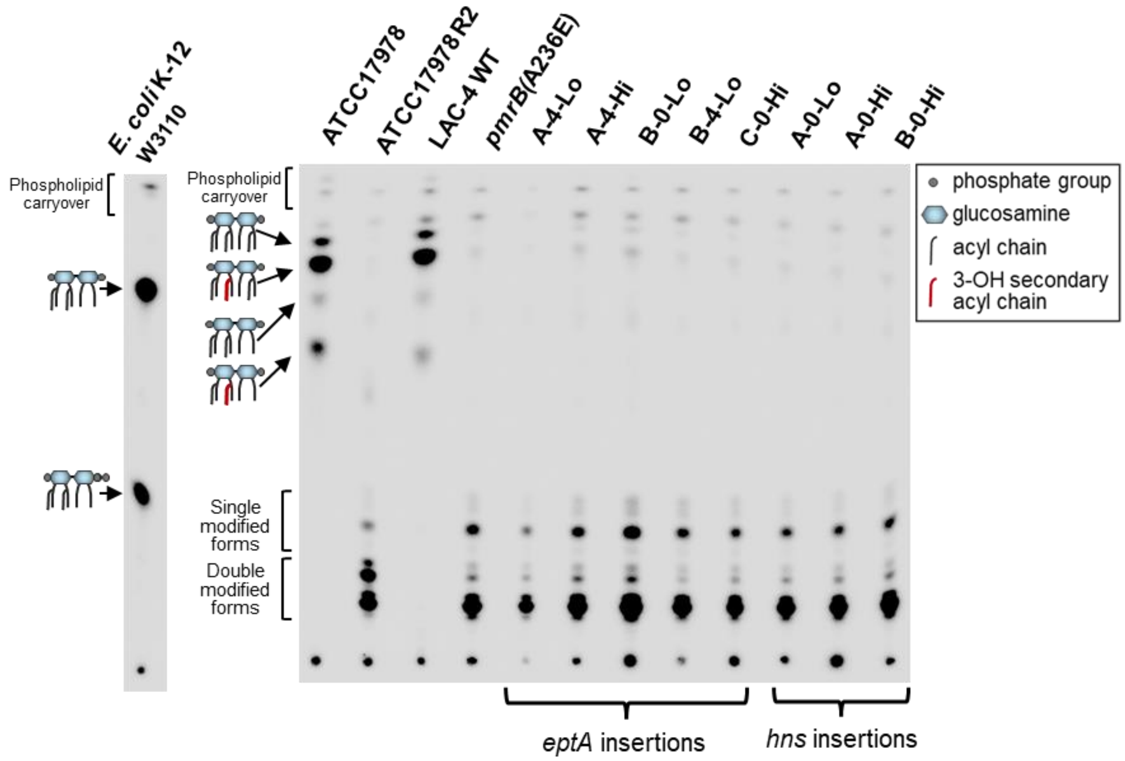

Fig. S5. Lipid A profile of representative *pmrB*(A236E) isolates with insertions in the vicinity of *eptA* and *hns*.

Lipid A profile of *pmrB* mutants. Lipid A from denoted strains was isolated, radiolabeled with ^{32}P and fractionated by TLC (Materials and Methods). Double-modified forms refer to the addition of phosphoethanolamine and/or galactosamine.

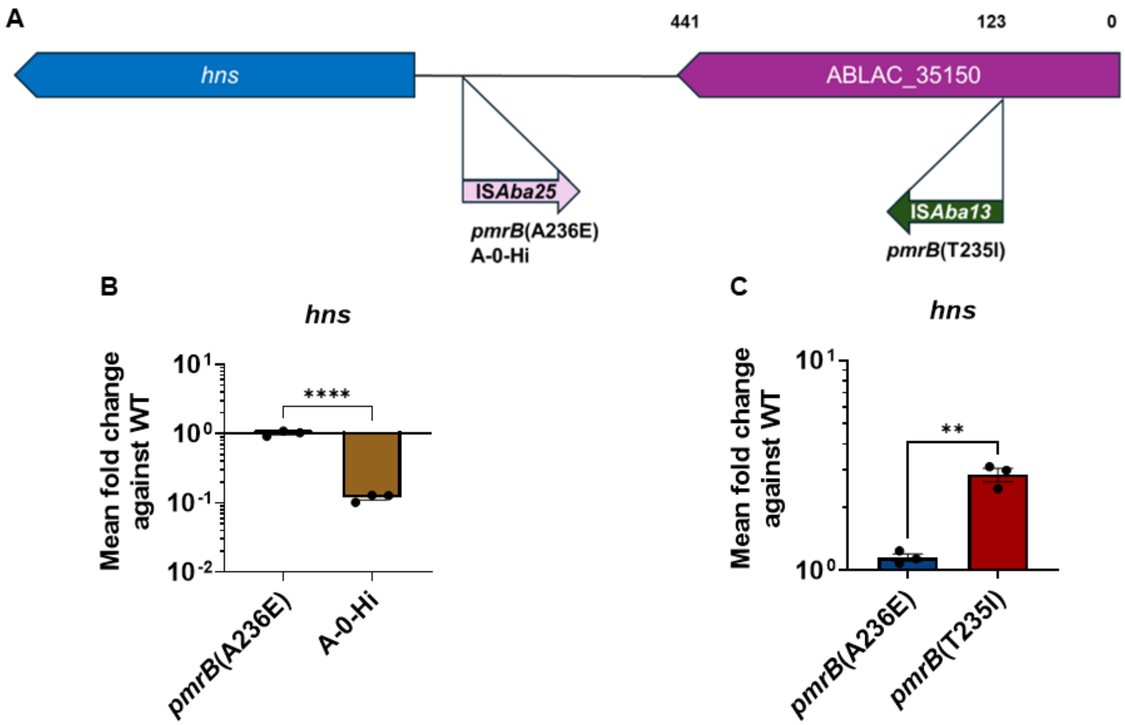

Fig. S6. IS*Aba* insertions upstream *hns* alters this gene's expression.

A. Location and orientation of IS*Aba* insertions upstream ABLAC_35140 (*hns*) in the *pmrB*(T235I) and the *pmrB*(A236E) A-0-Hi strains. Not drawn to scale. **B-C.** Expression of *hns* in denoted strains. Transcript levels of *hns* in mutants relative to WT were quantified via RT-qPCR. Mean $\pm$ SEM from three biological replicates shown. Statistical analysis of gene expression changes was performed using unpaired two-tailed t-tests. $^{**}P < 0.01$, $^{****}P < 0.0001$.

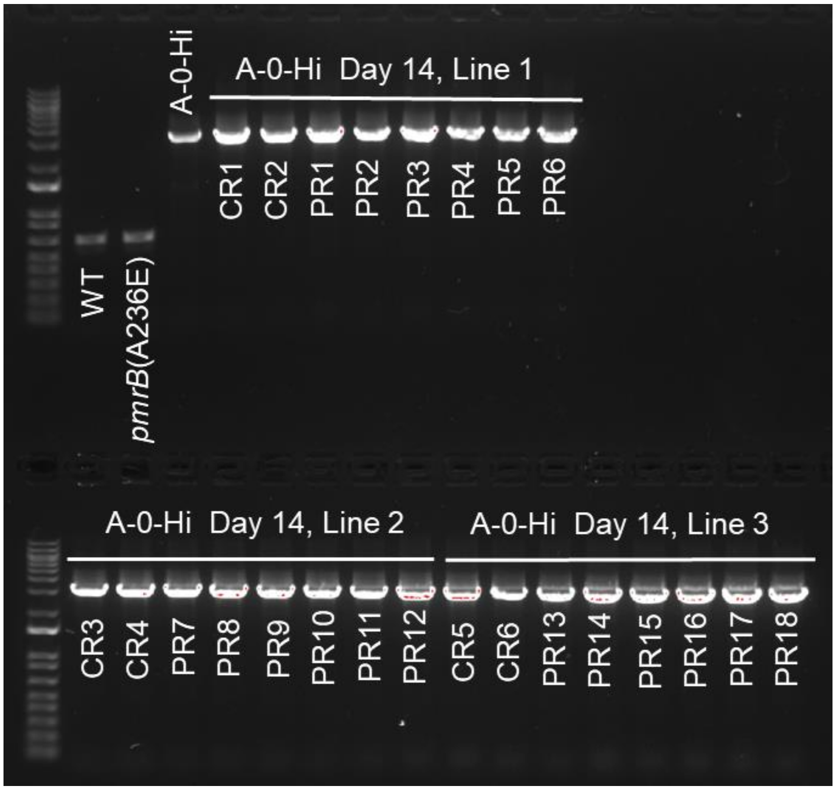

Fig. S7. *pmrB*(A236E) A-0-Hi pseudorevertants retain their insertion upstream *hns*. PCR amplification of the *hns* gene and its upstream region in denoted strains. After 14 days of antibiotic free passaging, 18 *pmrB*(A236E) A-0-Hi pseudorevertants (PR) were collected by picking colonies growing on LB plates and 6 colistin resistant (CR) colonies were collected from LB plates supplemented with 16µg/mL colistin (Fig. 6A). Colony PCR of the *hns* gene region was performed in denoted strains. The presence or absence of insertion mutations was visualized by running the PCR products on a 1% agarose TAE gel. WT and *pmrB*(A236E) were included as controls.

A

Strain:	A-0-Hi	PR1	PR7	PR8	PR13	PR14	PR15	PR18	WT
Doubling time ± SEM (min)	19.73 ± 0.16	19.38 ± 0.14 (ns)	19.16 ± 0.18 (ns)	20.19 ± 0.24 (ns)	19.08 ± 0.23 (ns)	19.02 ± 0.14 (ns)	18.77 ± 0.27 (*)	20.04 ± 0.18 (ns)	17.32 ± 0.04 (****)

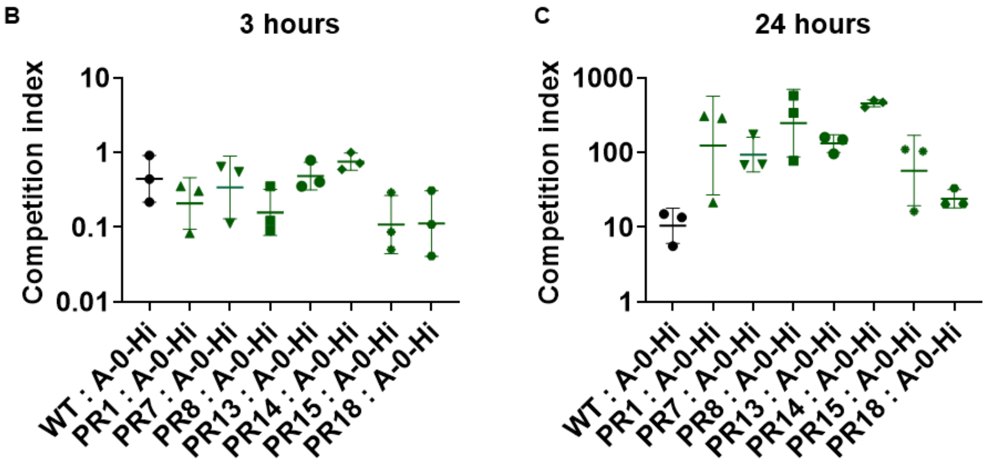

Fig. S8. A-0-Hi pseudorevertants outcompete their resistant parent when grown past exponential phase.

A. Growth rates from data shown in Figure 6D. Mean doubling time ± SEM of three biological replicates shown. Statistical analysis of doubling time was performed using One-way Anova followed by Dunnett's multiple comparison. **B-C.** Competition of pseudorevertants versus their *pmrB*(A236E) A-0-Hi parent in broth. Denoted strains were mixed at approximately 1:1 and inoculated into LB broth. The ratio of the colistin sensitive strains to their resistant parent was measured at hours 0, 3 and 24. Competition Index (CI) determined as described. Geometric Mean CI ± SD of 3 biological replicates shown.

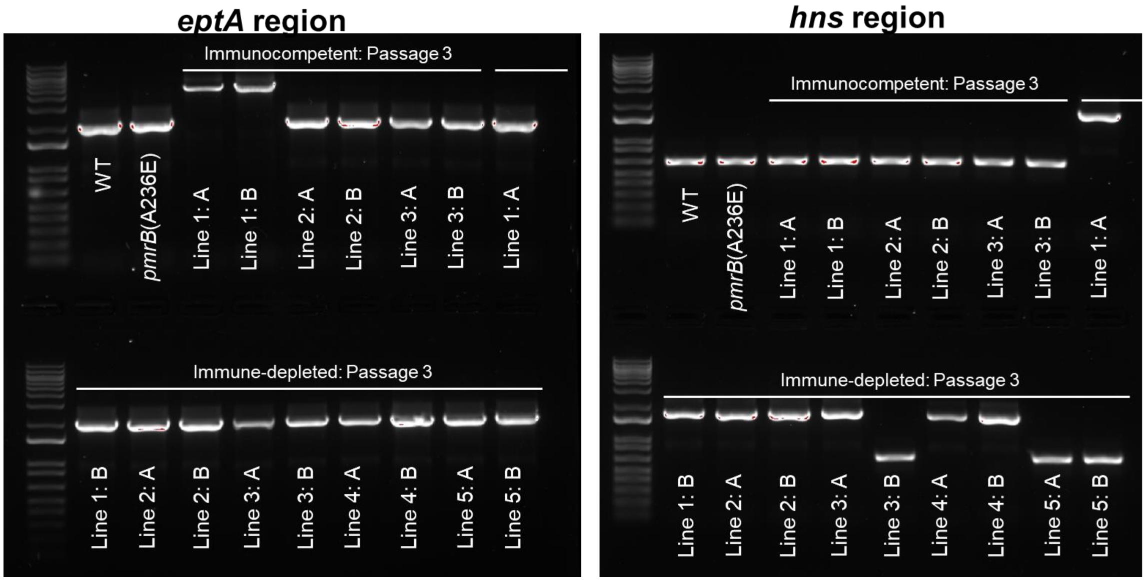

Fig. S9. Insertions in the vicinity of *eptA* and *hns* can be detected in *pmrB*(A236E) isolates passaged in mice treated with colistin.

Detection of insertions in the vicinity of *eptA* and *hns*. Frozen pools from passage 3 of the WT:*pmrB*(A236E) competitions in the presence of colistin treatment (Fig. 2D, E) were diluted and plated in LB plates supplemented with colistin 2µg/mL to select for *pmrB*(A236E) colonies and against WT. Two colonies per line (from both the immunocompetent and immune-depleted pools) growing at 2ug/mL colistin were re-streaked on identical colistin-containing plates. Colony PCR of both the *eptA* and *hns* gene regions was performed in denoted strains. The presence of new insertion mutations was visualized by running the PCR products on a 1% agarose TAE gel.

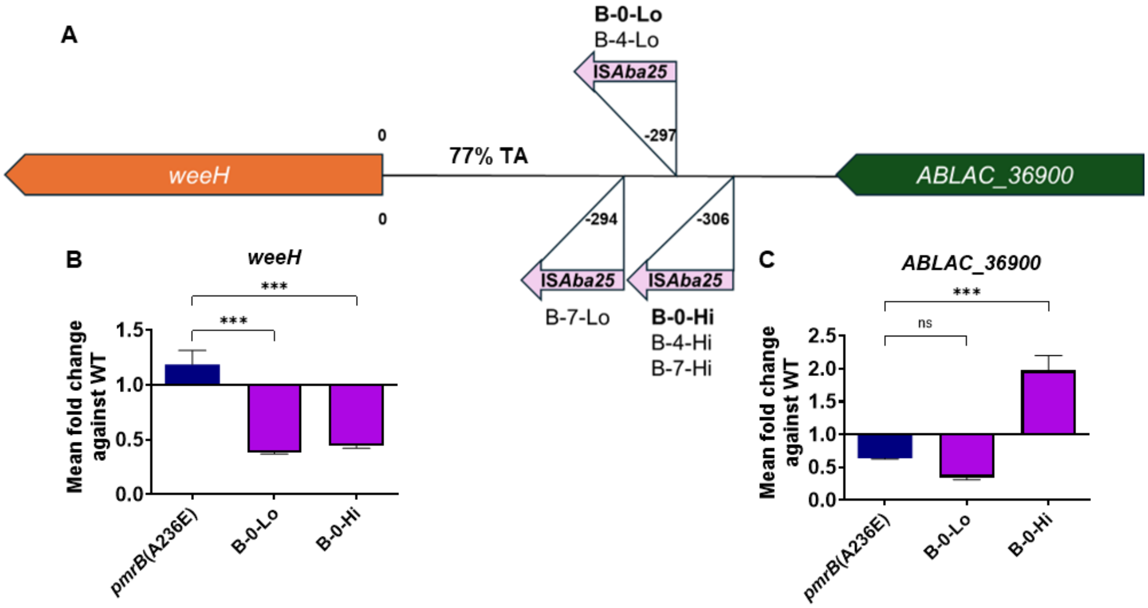

Fig. S10. IS*Aba* insertions located upstream of *weeH* reduce expression of downstream gene.

A. Location and orientation of IS*Aba* insertions upstream ABLAC_36890 (*weeH*) in colistin-enriched *pmrB*(A236E) isolates described in Figure 5. Representative isolates (in bold) were selected for further analysis. B-0-Lo contains an IS*Aba* insertion upstream *eptA* and B-0-Hi contains one in the *hns* gene. Not drawn to scale. **B-C.** Expression of genes flanking the IS*Aba* insertions upstream *weeH*. Transcript levels of *weeH* (ABLAC_36890) and ABLAC_36900 in mutants relative to WT was quantified via RT-qPCR. Mean $\pm$ SEM from three biological replicates shown**.** Statistical analysis of gene expression changes were performed using one-way Anova followed by Dunnett's multiple comparison.***$P < 0.001$; ns, not significant.

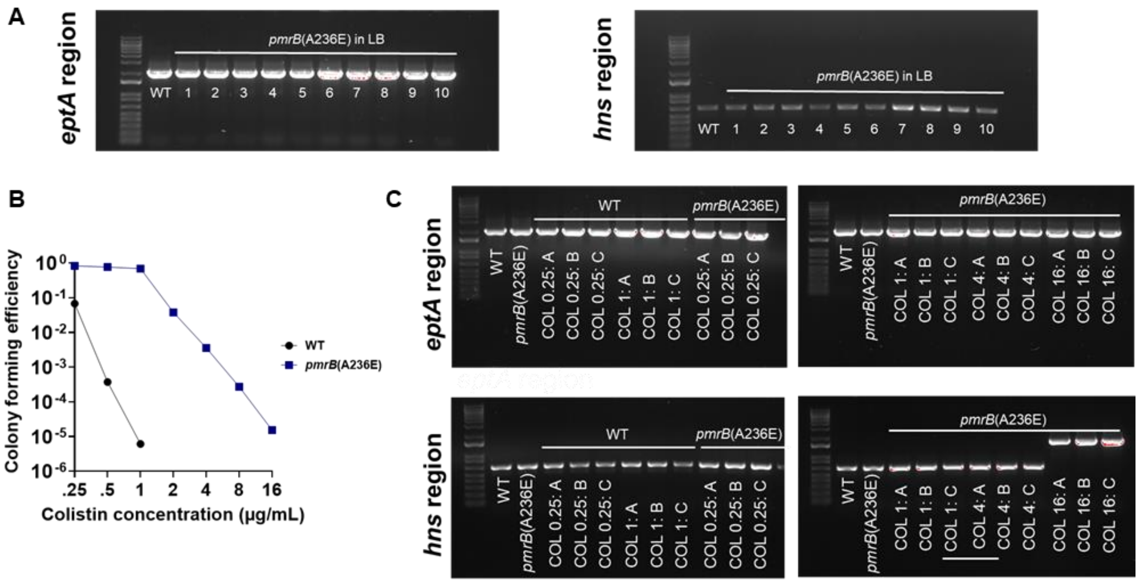

Fig. S11. Insertion mutations in the vicinity of *eptA* or *hns* are only detected in the *pmrB*(A236E) mutant when plated at high colistin concentrations.

A. The frozen stock of *pmrB*(A236E) was streaked on a LB plate. 10 individual colonies were randomly selected for colony PCR of the *eptA* and *hns* gene regions. **B.** Population analysis profiling of WT and *pmrB*(A236E)**.** Cultures of denoted strains are serially diluted and plated in LB supplemented with noted concentrations of colistin. Colony forming efficiency (CFE) was calculated as described. **C.** Colony PCR of the *eptA* and *hns* regions was performed on WT and *pmrB*(A236E) colonies selected at 0.25, 1, 4, and 16 µg/mL colistin (B). The presence or absence of insertion mutations was visualized by running the PCR products on a 1% agarose TAE gel.

**Class**	**Antibiotic**	**WT**	***pmrB*(T235I)**	***pmrB*(A236E)**
Carbapenems	Imipenem	2μg/mL	2μg/mL	2μg/mL
	Meropenem	2μg/mL	2μg/mL	2μg/mL
Cephalosporins	Ceftazidime	256μg/mL	**128μg/mL**	**128μg/mL**
Tetracyclines	Tigecycline	1μg/mL	1μg/mL	1μg/mL
	Tetracycline	2μg/mL	2μg/mL	2μg/mL

Table S1. Sensitivity of evolved *pmrB* mutants to clinically useful antibiotics.

MIC of denoted strains was obtained using the broth microdilution assay (Materials and Methods). Exponential phase cultures of denoted strains were grown to exponential phase and inoculated at A600 = 0.03 into 96-well plates with LB supplemented with 2x dilutions of antibiotics. Plates were incubated at 37°C with shaking and A600 was measured every 15mins. MIC refers to the lowest concentration of antibiotic tested in which no growth was observed.

Table S2. Strains used in this study.

Strain (clone name)	Description	Accession	COL MIC	Source	Relevant alleles
E. coli K12	*E. coli* W3110	-	-	(1)	-
ATCC17978	*A. baumannii* ATCC17978UN	-	-	(2)	-
ATCC17978 R2	Polymyxin-resistant ATCC17978UN	-	-	(2)	*pmrB*(T235I)
LAC-4 WT	Clinical isolate from Los Angeles county nosocomial outbreak.	SRX29091891, SRX29091953	0.38	(3)	-
ΔpABLAC2	Spontaneous plasmid loss resulting in Gentamycin sensitivity.	SRX29091892	0.38	This	*ΔpABLAC2*
pmrB(T235I) (COL17)	COL 4 µg/mL selection from immune-depleted frozen pool (Line A, Passage 16)	SRX29091903, SRX29091954	16	This	*pmrB*(T235I), ABLAC_16720::IS*Aba25*, ABLAC_23900::IS*Aba1*, ABLAC_35150::IS*Aba13*
pmrB(A236E) (COL23)	COL 2 µg/mL selection from Immunocompetent frozen pool (Line C, Passage 16)	SRX29091914, SRX29091955	0.75	This	*pmrB*(A236E)
*lpxA** (LOS$^{(-)}$)	LOS deficient LAC-4.	SRX29091925	8	This	*lpxA*(G142R)
lpxA pldA** (LB14)	LOS deficient LAC-4 selected at 42°C for improved growth.	SRX29091936	12	This	*lpxA*(G142R), *ΔpABLAC2*, *pldA*(Δ1bp 84/1152).
lpxA pldA* ponA^{ins}* (LB13)	LOS deficient LAC-4 selected at 42°C for improved growth.	SRX29091947	12	This	*lpxA*(G142R), *ΔpABLAC2*, *pldA*(Δ1bp 84/1152), *ponA*(+GAT 8/2466)
lpxA pldA* ponA** (LB14)	LOS deficient LAC-4 selected at 42°C for improved growth.	SRX29091958	12	This	*lpxA*(G142R), *ΔpABLAC2*, *pldA*(Δ1bp 84/1152), *ponA*(E147Q)
A-0-Lo (I1)	Derived from COL23. COL 2µg/mL selection from Line A, Day 0. Colony collected from COL 16µg/mL plate.	SRX29091969, SRX29091956	6	This	*pmrB*(A236E), new IS*Aba*
A-4-Lo (I2)	Derived from COL23. COL 2µg/mL selection from Line A, Day 4. Colony collected from COL 16µg/mL plate.	SRX29091977, SRX29091957	1	This	*pmrB*(A236E), new IS*Aba*

A-7-Lo (I3)	Derived from COL23. COL 2μg/mL selection from Line A, Day 7. Colony collected from COL 16μg/mL plate.	SRX29091893, SRX29091959	2	This	*pmrB*(A236E), *gacS*(310C), new IS*Aba*
B-0-Lo (I7)	Derived from COL23. COL 2μg/mL selection from Line B, Day 0. Colony collected from COL 16μg/mL plate.	SRX29091894, SRX29091960	2	This	*pmrB*(A236E), new IS*Aba*
B-4-Lo (I8)	Derived from COL23. COL 2μg/mL selection from Line B, Day 4. Colony collected from COL 16μg/mL plate.	SRX29091895, SRX29091961	1.5	This	*pmrB*(A236E), new IS*Aba*
B-7-Lo (I9)	Derived from COL23. COL 2μg/mL selection from Line B, Day 7. Colony collected from COL 16μg/mL plate.	SRX29091896, SRX29091962	6	This	*pmrB*(A236E), *clpS*(Q90*), *eptA*(R127H)
C-0-Lo (I13)	Derived from COL23. COL 2μg/mL selection from Line C, Day 0. Colony collected from COL 16μg/mL plate.	SRX29091897, SRX29091963	3	This	*pmrB*(A236E), new IS*Aba*
C-4-Lo (I14)	Derived from COL23. COL 2μg/mL selection from Line C, Day 4. Colony collected from COL 16μg/mL plate.	SRX29091898, SRX29091964	4	This	*pmrB*(A236E), *clpA*(A302V), new IS*Aba*
C-7-Lo (I15)	Derived from COL23. COL 2μg/mL selection from Line C, Day 7. Colony collected from COL 16μg/mL plate.	SRX29091899, SRX29091965	4	This	*pmrB*(A236E), new IS*Aba*
A-0-Hi (I4)	Derived from COL23. COL 4μg/mL selection from Line A, Day 0. Colony collected from COL 16μg/mL plate.	SRX29091900, SRX29091966, SRX29091976	6	This	*pmrB*(A236E), new IS*Aba*
A-4-Hi (I5)	Derived from COL23. COL 4μg/mL selection from Line A, Day 4. Colony collected from COL 16μg/mL plate.	SRX29091901, SRX29091967	4	This	*pmrB*(A236E), new IS*Aba*
A-7-Hi (I6)	Derived from COL23. COL 4μg/mL selection from Line A, Day 7. Colony collected from COL 16μg/mL plate.	SRX29091902, SRX29091968	4	This	*pmrB*(A236E), new IS*Aba*
B-0-Hi (I10)	Derived from COL23. COL 4μg/mL selection from Line B, Day 0. Colony collected from COL 16μg/mL plate.	SRX29091904, SRX29091970	6	This	*pmrB*(A236E), new IS*Aba*

B-4-Hi (I11)	Derived from COL23. COL 4µg/mL selection from Line B, Day 4. Colony collected from COL 16µg/mL plate.	SRX29091905, SRX29091971	6	This	*pmrB*(A236E), *clpS*(+AC 132/414), new IS*Aba*
B-7-Hi (I12)	Derived from COL23. COL 4µg/mL selection from Line B, Day 7. Colony collected from COL 16µg/mL plate.	SRX29091906, SRX29091972	8	This	*pmrB*(A236E), new IS*Aba*
C-0-Hi (I16)	Derived from COL23. COL 4µg/mL selection from Line C, Day 0. Colony collected from COL 16µg/mL plate.	SRX29091907, SRX29091973	1.5	This	*pmrB*(A236E), new IS*Aba*
C-4-Hi (I17)	Derived from COL23. COL 4µg/mL selection from Line C, Day 4. Colony collected from COL 16µg/mL plate.	SRX29091908, SRX29091974	1.5	This	*pmrB*(A236E), new IS*Aba*
C-7-Hi (I18)	Derived from COL23. COL 4µg/mL selection from Line C, Day 7. Colony collected from COL 16µg/mL plate.	SRX29091909, SRX29091975	2	This	*pmrB*(A236E), ABLAC_02920(+T: -45nt upstream), new IS*Aba*
COL26	COL 2 µg/mL selection from immunocompetent frozen pool (Line B, Passage 16)	SRX29091910	1.5	This	*pmrA*(M12R)
COL27	COL 4 µg/mL selection from immunocompetent frozen pool (Line B, Passage 16)	SRX29091911	-	This	*pmrA*(M12R)
COL28	COL 4 µg/mL selection from immunocompetent frozen pool (Line B, Passage 16)	SRX29091912	8	This	*pmrB*(T235I), ABLAC_24300(P131S)
COL29	COL 2 µg/mL selection from immune-depleted frozen pool (Line B, Passage 10)	SRX29091913	6	This	*pmrB*(T235I), ABLAC_12660(Δ1bp 819/1569)
COL30	COL 2 µg/mL selection from immune-depleted frozen pool (Line B, Passage 10)	SRX29091915	6	This	*pmrB*(T235I)
COL31	COL 4 µg/mL selection from immune-depleted frozen pool (Line B, Passage 10)	SRX29091916	6	This	*pmrB*(T235I), ABLAC_06150(T15K)
COL33	COL 1 µg/mL selection from immune-depleted frozen pool (Line C, Passage 10)	SRX29091917	0.19	This	ISAba125 amplification, *envZ*(+GCCTTC 1084/1344), ABLAC_25350 (Δ1bp 290/387)

COL34	COL 1 µg/mL selection from immune-depleted frozen pool (Line C, Passage 10)	SRX29091918	0.18	This	*lpxC*(+GAATAT 501/903)
LB1	LOS deficient LAC-4 selected at 42°C for improved growth.	SRX29091919	-	This	*lpxA*(G142R), *ΔpABLAC2*, *pldA*(Δ1bp 84/1152), *ponA*(+GAT 8/2466)
LB2	LOS deficient LAC-4 selected at 42°C for improved growth.	SRX29091920	-	This	*lpxA*(G142R), *ΔpABLAC2*, *pldA*(Δ1bp 84/1152), *ponA*(E147Q)
LB3	LOS deficient LAC-4 selected at 42°C for improved growth.	SRX29091921	-	This	*lpxA*(G142R), *ΔpABLAC2*, *pldA*(Δ1bp 84/1152)
LB4	LOS deficient LAC-4 selected at 42°C for improved growth.	SRX29091922	-	This	*lpxA*(G142R), *ΔpABLAC2*, *pldA*(Δ1bp 84/1152), *ponA*(E147Q)
LB5	LOS deficient LAC-4 selected at 42°C for improved growth.	-	-	This	*lpxA*(G142R) reverted to WT
LB6	LOS deficient LAC-4 selected at 42°C for improved growth.	SRX29091923	-	This	*lpxA*(G142R), *ΔpABLAC2*, *pldA*(Δ1bp 84/1152)
LB7	LOS deficient LAC-4 selected at 42°C for improved growth.	SRX29091924	-	This	*lpxA*(G142R), *msbA*(L161S), ABLAC_27930(K112*), ABLAC_09320(V54G), ABLAC_19710(K438N)
LB8	LOS deficient LAC-4 selected at 42°C for improved growth.	SRX29091926	-	This	*lpxA*(G142R), *ΔpABLAC2*, *pldA*(Δ1bp 84/1152)
LB9	LOS deficient LAC-4 selected at 42°C for improved growth.	SRX29091927	-	This	*lpxA*(G142R), *ΔpABLAC2*, *pldA*(Δ1bp 84/1152), ABLAC_25350(+TG 173/387)
LB10	LOS deficient LAC-4 selected at 42°C for improved growth.	SRX29091928	-	This	*lpxA*(G142R), *ΔpABLAC2*, *pldA*(Δ1bp 84/1152)
LB11	LOS deficient LAC-4 selected at 42°C for improved growth.	SRX29091929	-	This	*lpxA*(G142R), *ΔpABLAC2*, *pldA*(Δ1bp 84/1152),

					ABLAC_25350(+AA 55/387)
LB15	LOS deficient LAC-4 selected at 42°C for improved growth.	SRX29091930	-	This	*lpxA*(G142R), *ΔpABLAC2, pldA*(Δ1bp 84/1152), *ponA*(E147Q)
LB16	LOS deficient LAC-4 selected at 42°C for improved growth.	-	-	This	*lpxA*(G142R) reverted to WT
LB17	LOS deficient LAC-4 selected at 42°C for improved growth.	SRX29091931	-	This	*lpxA*(G142R), *ΔpABLAC2, pldA*(Δ1bp 84/1152), *ponA*(E147Q)
LB18	LOS deficient LAC-4 selected at 42°C for improved growth.	SRX29091932	-	This	*lpxA*(G142R), *ΔpABLAC2, pldA*(Δ1bp 84/1152), *ponA*(E147Q)
PR1	A-0-Hi pseudorevertant selected on LB after drug free passaging (Day 14, Line 1)	SRX29091933	ColS	This	*pmrB*(Δ3bp: 1,206/1,335 nt)
PR2	A-0-Hi pseudorevertant selected on LB after drug free passaging (Day 14, Line 1)	SRX29091934	ColS	This	*pmrB*(Δ3bp: 1,206/1,335 nt)
PR3	A-0-Hi pseudorevertant selected on LB after drug free passaging (Day 14, Line 1)	SRX29091935	ColS	This	*pmrB*(Δ3bp: 1,206/1,335 nt)
PR4	A-0-Hi pseudorevertant selected on LB after drug free passaging (Day 14, Line 1)	SRX29091937	ColS	This	*pmrB*(Δ3bp: 1,206/1,335 nt)
PR5	A-0-Hi pseudorevertant selected on LB after drug free passaging (Day 14, Line 1)	SRX29091938	ColS	This	*pmrB*(Δ3bp: 1,206/1,335 nt)
PR6	A-0-Hi pseudorevertant selected on LB after drug free passaging (Day 14, Line 1)	SRX29091939	ColS	This	*pmrB*(Δ3bp: 1,206/1,335 nt)
PR7	A-0-Hi pseudorevertant selected on LB after drug free passaging (Day 14, Line 2)	SRX29091940	ColS	This	*pmrB*(IS*Abe18*^{ins}: 163/1,335 nt)
PR8	A-0-Hi pseudorevertant selected on LB after drug free passaging (Day 14, Line 2)	SRX29091941	ColS	This	*pmrB*(G339V*)
PR9	A-0-Hi pseudorevertant selected on LB after drug free passaging (Day 14, Line 2)	SRX29091942	ColS	This	*pmrB*(G339V*)
PR10	A-0-Hi pseudorevertant selected on LB after drug free passaging (Day 14, Line 2)	SRX29091943	ColS	This	*pmrB*(G339V*)

PR11	A-0-Hi pseudorevertant selected on LB after drug free passaging (Day 14, Line 2)	**SRX29091944**	ColS	This	*pmrB*(G339V*)
PR12	A-0-Hi pseudorevertant selected on LB after drug free passaging (Day 14, Line 2)	**SRX29091945**	ColS	This	*pmrB*(G339V*)
PR13	A-0-Hi pseudorevertant selected on LB after drug free passaging (Day 14, Line 3)	**SRX29091946**	ColS	This	*pmrB*(Δ1bp: 1,299/1,335 nt)
PR14	A-0-Hi pseudorevertant selected on LB after drug free passaging (Day 14, Line 3)	**SRX29091948**	ColS	This	*pmrB*(Δ1bp: 1,299/1,335 nt)
PR15	A-0-Hi pseudorevertant selected on LB after drug free passaging (Day 14, Line 3)	**SRX29091949**	ColS	This	*pmrB*(Δ2bp: 1,121/1,335 nt)
PR16	A-0-Hi pseudorevertant selected on LB after drug free passaging (Day 14, Line 3)	**SRX29091950**	ColS	This	*pmrB*(Δ1bp: 1,299/1,335 nt)
PR17	A-0-Hi pseudorevertant selected on LB after drug free passaging (Day 14, Line 3)	**SRX29091951**	ColS	This	*pmrB*(Δ1bp: 1,299/1,335 nt)
PR18	A-0-Hi pseudorevertant selected on LB after drug free passaging (Day 14, Line 3)	**SRX29091952**	ColS	This	*pmrA*(IS*Aba25*^{ins}: 621/1,335 nt)
pmrB(T235I) BC1	The *pmrB*(T235I) allele was backcrossed from COL17 into the WT strain.		4	This	*pmrB*(T235I)
pmrB(A236E) BC1	The *pmrB*(A236E) allele was backcrossed from COL23 into the WT strain.		0.5	This	*pmrB*(A236E)

Table S3. Primers and Plasmids used in this study.

Primers	Sequence	Reference
**qRT_PCR primers**		
16s_qRT_For	CAGCTCGTGTCGTGAGATGT	(4)
16s_qRT_Rev	CGTAAGGGCCATGATGACTT	(4)
pmrC_qRT_For	CCATTTGGCTAGGTGCAATTT	This
pmrC_qRT_Rev	ACCGCATAATAGGTAGCAACAA	This
naxD_qRT_For	ACCGATCAATACCCCTCATT	(5)
naxD_qRT_Rev	GTTCGCCATAATTGGTCAGT	(5)
eptA_qRT_For	TCTATCTGGCTAGGTTTATTTCTG	This
eptA_qRT_Rev	CTATTAAAATAACTAATGTCGCCCC	This
hns_qRT_For	CGGTAACGTGGTTCTACAGATTTA	This
hns_qRT_Rev	AAAGATCAAGCAATCGAAGATGC	This
weeH_qRT_For	GGATTACCCTGTGCATCAAATG	This
weeH_qRT_Rev	TGGGTAGCATATAAGGTTCGTAAA	This
ABLAC_36900_qRT_For	CCAAGGAGAGCAATAAGCAAAC	This
ABLAC_36900_qRT_Rev	GTGGTGGTGGAGTAGCAATAG	This
**Endpoint PCR**		
hns_region_For	CGCTTGGCTTTTTAAGAGG	This
hns_region_Rev	TACAGGTGAAGCAGTTATGG	This
eptA_region_For	TCAACTGGGGATAACTATGC	This
eptA_region_Rev	GGCTCTAGATTACTCGAACC	This
pmrCAB_For	GTTTCAATTTCTACATTAAAGC	This
pmrCAB_Rev	TATTCATCGTTTTGAGTTC	This
ABLAC_35150_region_F	ATCGCTTGGCTTTTTAAGAG	This
ABLAC_35150_region_R	TACGACCAGAAAGTAGCATG	This
ABLAC_16720_region_F	GATGTATGAAGTCCGTCCAG	This
ABLAC_16720_region_R	AATATCTTTGGTAGCAGCCC	This
ABLAC_23900_region_F	TGAGTTTCTCTACACGCTTC	This
ABLAC_23900_region_R	CTTATGTTGTTACCTCCTGC	This
**Plasmid construction primers**		
pmrB_Fw_NotI_800	GACTCAGCGGCCGCAGTCAGGATAATTTATT	This
pmrB_BamHI_Rv_800	CAGTCAGGATCCCTCGTCGCTTTTTTAT	This

Plasmids	Description	Reference
pJB4648APRA	derived from pSR47S. *oriTRP4 oriR6K sacB ApraR*	(6)
pJB4648APRA235-2-4	pJB4648-NotI-*pmrBT235I*-BamHI	This
pJB4648APRA236-6-3	pJB4648-NotI-*pmrBA236E*-BamHI	This

Dataset Legends

Dataset S1 (separate file). Whole genome sequencing of mutants with altered colistin susceptibility in passaged pools.

Isolates with altered colistin sensitivity able to grow on their respective colistin plates were saved and sequenced (Fig. S1). Colistin MIC of these isolates was determined via E-test strips (Materials and Methods). Mutations were detected by whole genome sequencing followed by BRESEQ analysis (Materials and Methods).

Dataset S2 (separate file). Mutations present in LOS deficient suppressors selected at 42°C.

LOS deficient isolates selected at 42°C in experiments described in Figure 3A were whole genome sequenced. Mutations were detected as described (Materials and Methods).

Dataset S3 (separate file). Detection of IS*Aba* insertions in *pmrB*(A236E) isolates after selection for high colistin resistance.

PacBio long-read sequencing was performed on denoted strains. Sequencing and alignment to LAC-4 chromosome performed as described (Materials and Methods). Identity of newly transposed IS*Aba* insertion sequences, their location and directionality are noted. The TA% of the genes and intergenic regions of insertion sites are noted.

Dataset S4 (separate file). Mutations detected via the BRESEQ pipeline.

Illumina short-read sequencing was performed on denoted strains. The BRESEQ pipeline was used to align reads to the reference genome and detect mutations. Only the mutations present in the evolved LAC-4 isolates and not their WT parent are shown. 100% means that all sequenced reads support the presence of the denoted mutation.

SI References

1. L. M. VanOtterloo, L. A. Macias, M. J. Powers, J. S. Brodbelt, M. S. Trent, Characterization of Acinetobacter baumannii core oligosaccharide synthesis reveals novel aspects of lipooligosaccharide assembly. *mBio* **15**, e03013-23 (2024).

2. L. A. Arroyo, *et al.*, The pmrCAB operon mediates polymyxin resistance in Acinetobacter baumannii ATCC 17978 and clinical isolates through phosphoethanolamine modification of lipid A. *Antimicrobial agents and chemotherapy* **55**, 3743–51 (2011).

3. S. C. Valentine, *et al.*, Phenotypic and molecular characterization of Acinetobacter baumannii clinical isolates from nosocomial outbreaks in Los Angeles County, California. *Journal of clinical microbiology* **46**, 2499–507 (2008).

4. W. Huo, *et al.*, Immunosuppression broadens evolutionary pathways to drug resistance and treatment failure during Acinetobacter baumannii pneumonia in mice. *Nat Microbiol* **7**, 796–809 (2022).

5. C.-Y. Chin, K. A. Gregg, B. A. Napier, R. K. Ernst, D. S. Weiss, A PmrB-Regulated Deacetylase Required for Lipid A Modification and Polymyxin Resistance in Acinetobacter baumannii. *Antimicrobial agents and chemotherapy* **59**, 7911–4 (2015).

6. H. L. Andrews, J. P. Vogel, R. R. Isberg, Identification of Linked Legionella pneumophila Genes Essential for Intracellular Growth and Evasion of the Endocytic Pathway. *Infection and Immunity* **66**, 950–958 (1998).
